# Controls on Coral-Ground Development along the Northern Mesoamerican Reef Tract

**DOI:** 10.1371/journal.pone.0028461

**Published:** 2011-12-14

**Authors:** Rosa E. Rodríguez-Martínez, Adán G. Jordán-Garza, Miguel A. Maldonado, Paul Blanchon

**Affiliations:** 1 Unidad de Sistemas Arrecifales, Instituto de Ciencias del Mar y Limnología, Universidad Nacional Autónoma de México, Cancún, Quintana Roo, México; 2 Centro Ecológico Akumal A.C. Akumal, Quintana Roo, México; Biodiversity Insitute of Ontario - University of Guelph, Canada

## Abstract

Coral-grounds are reef communities that colonize rocky substratum but do not form framework or three-dimensional reef structures. To investigate why, we used video transects and underwater photography to determine the composition, structure and status of a coral-ground community located on the edge of a rocky terrace in front of a tourist park, Xcaret, in the northern Mesoamerican Reef tract, Mexico. The community has a relatively low coral, gorgonian and sponge cover (<10%) and high algal cover (>40%). We recorded 23 species of Scleractinia, 14 species of Gorgonacea and 30 species of Porifera. The coral community is diverse but lacks large coral colonies, being dominated instead by small, sediment-tolerant, and brooding species. In these small colonies, the abundance of potentially lethal interactions and partial mortality is high but decreases when colonies are larger than 40 cm. Such characteristics are consistent with an environment control whereby storm waves periodically remove larger colonies and elevate sediment flux. The community only survives these storm conditions due to its slope-break location, which ensures lack of burial and continued local recruitment. A comparison with similar coral-ground communities in adjacent areas suggests that the narrow width of the rock terrace hinders sediment stabilization, thereby ensuring that communities cannot escape bottom effects and develop into three-dimensional reef structures on geological time scales.

## Introduction

It is widely considered that reefs are shaped by the dynamic interaction between accretion and erosion and develop geological structures only where the calcification rate exceeds erosion [1], [2]. But for poorly known reasons, communities dominated by Scleractinian corals do not always produce reefs with geological framework structures and have consequently been referred to as non-reef or non-framework building coral communities [3], [4], [5], coral carpets [3] or coral-grounds [6]. Their species compositions are similar to framework-building coral reefs but usually consist of small scattered colonies growing directly on bedrock. Yet hard-coral cover in some of these communities can be 50% or higher [4].

Such non-accretional communities have been reported from latitudinally ‘marginal’ areas where conditions are close to the environmental thresholds for coral survival [7], [8], or from localized areas affected by environmental conditions that are widely accepted as suboptimal [5], [9], [10]. Reports of specific environmental factors limiting framework development are numerous, including proximity to upwelling areas or groundwater outflow [7], high wave exposure [11], [12], temperature extremes [7], [13], low aragonite saturation [7], high sediment flux or absence of topographic shelter from sedimentation [4], [5], [7] and high rates of bioerosion [14], [15]. However, unfavorable conditions for reef accretion are much more widespread than these local or marginal areas, and exist in almost all reef systems. If this were not the case, reefs would develop as continuous breakwaters that lacked discontinuities.

Here we investigate a non-reefal coral-ground community from Xcaret, in the northern section of the Mesoamerican Reef where reef-tracts are well developed but discontinuous [6]. This region lacks surface rivers due to the highly porous and permeable limestone of the Yucatan Peninsula and marine conditions are therefore uniform and generally well-suited for coral reef development [6]. We report the composition and structure of this coral-ground community and determine its status in terms of disease prevalence, competitive interactions, and partial mortality. Using these data, we examine the potential processes responsible for preventing the survival and continuous growth of coral colonies at these sites and consider why three-dimensional reef structures are absent.

## Results

For the purpose of this work, we define a coral-ground as a rocky substratum colonized by multispecies assemblages of Scleractinian corals, sponges, and gorgonians, which do not accrete to form a framework or three-dimensional structures [6]. The coral-ground assemblage on the shallow rocky terrace off Xcaret is composed of Scleractinian corals (bottom cover = 5.9%±2.2%), gorgonians (7.4%±7.0%), encrusting and erect sponges (3.6%±1.4%), macroalgae (16.6%±8.3%) and turf algae (62.6%±13.4%). Abiotic substrata (0.7%±1.1%) consisted of rock pavement and skeletal sand and gravel. Recently dead coral cover was low (0.3%±0.3%). Overall, we recorded 23 species of Scleractinia (Table 1), 30 of Porifera, 14 of Gorgonacea, four of Actinaria, two of Milleporina, two of Zoanthiniaria, one of Stylasterina and one of Chordata (Table 2). The dominant macroalgae were *Dictyota*, *Halimeda*, *Penicillus* and *Riphocephallus*.

**Table 1:** Number of coral colonies (n), colony size (maximum diameter in cm), percent colony mortality and juvenile (colonies <5 cm) contribution to the total number of colonies of each species in the coral-ground assemblage of Xcaret in 2005.

Species	Code	n	Diameter (cm)	Partial mortality (%)	Juvenile
			mean (± SD)	mean (± SD)	contribution (%)
*Acropora cervicornis*	Acer	3	21.0 (1.7)	8.9 (15.4)	-
*Agaricia agaricites*	Aaga	255	9.2 (5.5)	10.2 (17.7)	22.7
*Agaricia fragilis*	Afra	1	11.1 (-)	0	-
*Agaricia humilis*	Ahum	3	11.2 (5.1)	0	-
*Agaricia tenuifolia*	Aten	108	13.1 (10.9)	4.0 (11.3)	9.3
*Diploria clivosa*	Dcli	1	30.8 (-)	0	-
*D. labyrinthiformis*	Dlab	9	26.0 (11.4)	5.6 (8.4)	-
*D. strigosa*	Dstr	104	14.7 (9.8)	11.2 (16.3)	21.2
*Dichocoenia stokesii*	Dsto	72	8.5 (4.4)	23.7 (27.6)	29.2
*Isophyllastrea rigida*	Irig	3	4.9 (3.9)	6.4 (11.2)	66.7
*Leptoseris cucullata*	Lcuc	4	10.0 (5.8)	0	-
*Madracis decactis*	Mdec	22	5.4 (3.6)	23.7 (27.6)	50.0
*Meandrina meandrites*	Mmea	13	16.3 (9.5)	6.6 (10.8)	15.4
*Montastraea annularis*	Mann	5	14.7 (14.4)	35.5 (40.0)	20.0
*M. faveolata*	Mfav	22	13.4 (6.9)	27.1 (26.5)	9.1
*M. cavernosa*	Mcav	64	15.6 (10.2)	23.8 (29.6)	10.9
*Porites astreoides*	Past	161	5.9 (3.5)	12.0 (19.3)	47.8
*P. divaricata*	Pdiv	41	5.1 (4.1)	9.5 (19.2)	63.4
*P. furcata*	Pfur	6	8.1 (4.2)	7.6 (8.4)	33.3
*P. porites*	Ppor	94	10.5 (9.0)	8.8 (16.6)	30.9
*Siderastrea radians*	Srad	10	2.6 (1.1)	0	90.0
*S. siderea*	Ssid	556	7.6 (7.0)	22.4 (23.7)	52.0
*Stephanocoenia intercepta*	Sint	24	4.8 (3.5)	18.6 (26.7)	66.7
**Total**		**1581**	**9.2 (6.0)**	**15.8 (22.2)**	**65.6**

**Table 2:** List of non-scleractinian benthic fauna recorded in Xcaret in 2005.

**Phylum CNIDARIA**	**Phylum PORIFERA**
**Clase ANTHOZOA**	**Clase DEMOSPONGIAE**
**Orden ACTINARIA**	**Aiolochroia crassa**
*Condylactis gigantea*	**Agelas conifera**
*Lebrunia danae*	**Agelas dispar**
*Bartholomea annulata*	**Aka brevitubulata**
*Stichodactyla helianthus*	*Aka coralliphaga*
	**Amphimedon complanata**
**Orden GORGONACEA**	**Amphimedon compressa**
*Briareum asbestinum*	*Aplysina cauliformis*
*Eunicea calyculata*	*Aplysina fistularis*
*Eunicea laciniata*	**Aplysina lacunosa**
*Eunicea mammosa*	*Callyspongia plicifera*
*Eunicea tourneforti*	**Callyspongia vaginalis**
*Gorgonia flabelum*	**Cinachyrella alloclada**
*Muricea atlantica*	**Cliona delitrix**
*Muricea muricata*	**Cliona varians**
*Plexaura flexuosa*	**Desmapsamma anchorata**
*Plexaura homomalla*	**Ectyoplasia ferox**
*Plexaurella dichotoma*	**Geodia neptuni**
*Pseudoplexaura porosa*	*Iotrochota birotulata*
*Pseudopterogorgia americana*	*Ircina felix*
**Pseudopterogorgia rigida**	*Ircina strobilina*
	**Mycale laevis**
***Orden MILLEPORINA***	**Myrmekioderma gyroderma**
*Millepora alcicornis*	*Niphates digitalis*
*Millepora complanata*	**Niphates erecta**
	**Oceanapia bartschi**
***Orden STYLASTERINA***	**Plakortis angulospiculatus**
*Stylaster roseus*	**Verongula gigantea**
	Xetospongia muta*
**Orden ZOANTHINIARIA**	
*Palythoa caribaeorum*	*Phylum CHORDATA*
*Zoanthus sociatus*	*Clase ASCIDIACEA*
	**Trididemnum solidum**

Species encountered outside the transects are included.

A total of 1581 colonies of Scleractinian coral species were recorded, with massive growth-forms being the most common (83.8% of the total). Overall, mean colony density was 5.3 colonies m^−2^ (±2.6). Three coral species were dominant and contributed 61.4% of the total number of colonies recorded in the transects: *Siderastrea siderea* (36.9% of the colonies; density = 2.0±0.9 col m^−2^), *Agaricia agaricites* (14.4%; density = 1.0±0.7 col m^−2^) and *Porites astreoides* (10.5%; density = 0.6±0.3 col m^−2^). Subordinate species included *Diploria strigosa* (7.6%), *Dichocoenia stokesii* (6.0%), *A. tenuifolia* (5.7%) and *Montastraea cavernosa* (4.7%). Key reef-building species of the of the *Montastraea annularis* species complex were present but relatively rare (1.7%), *Acopora palmata* was not observed on the sampled transects and only three colonies of *A. cervicornis* were present (Table 1).

**Figure 1:**
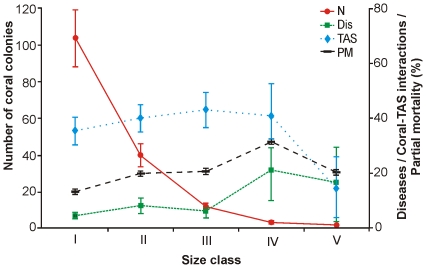
Coral colonies, diseases (%), coral-TAS mats competitive interactions and partial mortality in relation to size. Number of coral colonies (N), percentage of colonies with disease signs (Dis) and involved in a competitive interaction with turf-algal sediment mats (TAS) and percent colony mortality (PM) per transect (N = 10) in relation to size class (I: 0 to 10 cm diameter, n = 1037 colonies, II: >10 to 20 cm, n = 400 colonies, III: >20 to 30 cm, n = 104 colonies, IV: >30 to 40 cm, n = 28 colonies, V: >40 cm, n = 12 colonies) at Xcaret in 2005. Means ± Standard Error.

The size-frequency distribution of the coral colonies was strongly skewed, with >90% of the colonies smaller than 20 cm (Figure 1) and with only three colonies larger than 50 cm in diameter. The average diameter of coral colonies was 9.2 cm (±7.9). A few relatively large (>1 m) colonies of *A. palmata* and *M. annularis* species complex were observed in the study area but outside the belt-transects. Of the 23 coral species recorded, 17 had at least one juvenile (≤5 cm diameter) within the transects (Table 1). Overall, 36.9% of the coral colonies were juveniles (≥0.6 cm and ≤5 cm in diameter) and had an average age of 1.5±0.5 years (range: 0.3–2.5 years), assuming their growth rates in diameter were on average 2.02 cm yr-^−1^ (±0.68) [16]. Of the juveniles in the assemblage, 36% belonged to brooding species, while 49% belonged to *S. siderea*, a broadcast spawner.

Given the uniformity in size between the coral species, we pooled them into 10 cm size-classes in order to examine the relation between colony size and: (a) number of colonies, (b) partial mortality, (c) coral diseases, and (d) competitive interactions with TAS mats (Figure 1). The number of coral colonies was significantly different between size classes (One way ANOVA F_4_ = 68, p<0.001). A post-hoc analyses (Tukey's HSD) showed significant differences in all classes (p<0.05) except between IV and V (p = 0.68).

Partial mortality of coral colonies was recorded in 18 of the 23 coral species and the mean values ranged from 4 to 36% (Table 1). Overall, partial mortality was significantly different between size classes (F_4_ = 254, p<0.001, Figure 1), with coral colonies in the smallest size class (<10 cm) having significantly lower mean values (13.3±0.5%; Tukey's HSD, p<0.02) than those in the other size classes (mean range: 19.9–31.5%), except for the largest size class (p = 0.86). The highest values of partial mortality (31.5±0.7%) were recorded in the 30–40 cm size-class (class IV; Figure 1). At the species level, the highest percentages were recorded in *M. annularis* (mean = 36%), *M. faveolata* (27%), *M. cavernosa* (24%), *Madracis decactis* (24%), *D. stokesii* (24%) and *S. siderea* (22%) (Table 1).

In terms of coral diseases, 4.7% of the colonies had dark-spot disease, 0.4% had yellow-band disease, 0.2% had white-plague disease and 1.4% had tissue necrosis (Table 3). Mean prevalence of coral diseases was relatively low (<10%) in colonies smaller than 30 cm in diameter (Classes I–III) and increased to about 20% as colonies grew (Figure 1). Differences in disease prevalence between size classes were not statistically significant (Kruskal-Wallis test H_(4,45)_ = 7.63, p = 0.10) due to the high variability recorded between transects (Figure 1). The highest disease prevalence values per species were recorded in *M. faveolata*, where 27.3% of the colonies had yellow-band disease, *S. siderea*, where 12.5% of the colonies had dark-spot disease, and *Porites divaricata*, where 12.2% of the colonies had tissue necrosis (Table 3).

**Table 3:** Percentage of Scleractinian colonies affected by diseases (Dis) and involved in competitive interactions (CI) in Xcaret in 2005.

	Dis (%)				CI (%)					
Code	DS	WP	YB	Nec	TAS	Malg	CCA	Falg	Gorg	Spo
Species affected (n)	5	1	2	9	11	13	14	9	10	12
Colonies affected (%)	4.7	0.4	0.2	1.4	33.3	11.7	4.3	1.3	1.7	4.0
Dominant species										
Aaga	1.6			1.2	19.2	13.3	3.5		1.6	3.1
Aten		0.9		0.9	0.9	5.6	4.6	1.9	0.9	1.9
Dsto				1.4	48.6	12.5	6.9		1.4	1.4
Dstr					24.0	21.2	2.9	1.0	3.8	10.6
Mcav	3.1				29.7	17.2	15.6	4.7	1.6	9.4
Mdec					40.9					
Mfav			27.3		27.3	27.3	4.5	4.5	4.5	13.6
Past				1.9	27.3	6.8	3.7	1.2	1.9	6.2
Pdiv				12.2		7.3	2.4			2.4
Ppor				4.3	2.1	6.4	7.4	5.3		2.1
Sint				4.2	37.5		4.2	4.2		
Ssid	12.1	0.4		0.5	59.0	12.6	2.9	0.7	1.8	2.7

Only species with more than 20 colonies are shown. WP: white-plague disease, DS: dark-spot disease, YB: yellow-band disease, Nec: necrosis, TAS: turf-algal sediment mats, Malg: macroalgae, CCA: calcareous coralline algae, Falg: filamentous algae, Gorg: gorgonian, Spo: sponges. Species codes and sample size as in Table 1.

Over 45% of the coral colonies had a competitive interaction with algae, especially with turf-algae sediment (TAS) mats (33.3% of the colonies) and macro-algae (11.7%) (Table 3). Coral interactions with TAS mats were recorded in eleven species at the base of colonies and at numerous points on the colony surface. The mean percentage of coral colonies involved in an interaction with TAS mats was significantly different between size classes (Kruskal-Wallis test H _(4, 45)_ = 19, p = 0.0008), with significantly less coral colonies being involved in an interaction in class V (14.3±11.8%) than in classes I, II and III (mean range: 35.5–43.0%, Figure 1), based on post-hoc multiple comparisons test (p<0.01). TAS mats were particularly abundant in *S. siderea* (59% of the colonies), *D. stokesii* (49%), *M. decactis* (41%) and *Stephanocoenia intercepta* (38%) (Table 3). Competitive interactions between corals and macro-algae and sponges were higher in coral colonies with massive forms, particularly in *M. faveolata* and *D. strigosa* (Table 3).

## Discussion

The low coral cover at Xcaret results from the rareness of large colonies and the relatively high abundance of small colonies. The average diameter of coral colonies (9.2 cm) was less than one third of the average recorded on the entire Mesoamerican Barrier Reef (33 cm [17]). With the exception of *S. siderea*, a spawner with adults that can reach large sizes, the abundant small colonies are mainly composed by species that are naturally small (<50 cm) and brood their larvae, such as *A. agaricites* and *P. astreoides* [18]. Other key reef-building coral species are present at Xcaret, but have low abundances and rarely exceed 80 cm in diameter, such as *M. annularis* species complex and *Acropora* spp. The rareness of large (>40 cm diameter) corals, alive or dead, implies that colonies may be selectively removed once they reach a certain size.

In addition to the absence of large corals, Xcaret's assemblage is dominated by sediment-tolerant species, such as *P. astreoides* and *S. siderea* [19], [20], therefore implicating sediment flux as a control. High sediment flux also explains the major cause of coral tissue death, encroachment by turf-algae sediment (TAS) mats. TAS mats are known to flourish in areas of high sedimentation and to be able to encroach a coral colony at a rate of 70 cm^2^ yr^−1^ [21], [22].

Even in the absence of high sediment flux, mortality in juvenile corals is known to be high regardless of species composition due to their inherent susceptibility to adverse interactions like predation or biological disturbance [23], [24], [25], [26]. However, our data show that as Xcaret corals grow larger than 40 cm, the proportion of competitive interactions, particularly with TAS mats, and partial mortality of colonies, diminish indicating that colonies escape deleterious bottom effects [27]. Coral diseases, which have increased considerably on Mexican Caribbean reefs in the last two decades [6], [22], do not appear to play a major role in the dynamics of Xcaret's coral assemblage, as their prevalence is low and similar in all size classes. The high abundance of juvenile colonies in the majority of species present allows for high population turnover and the maintenance of a relatively diverse coral assemblage on the Xcaret coral-ground.

**Figure 2:**
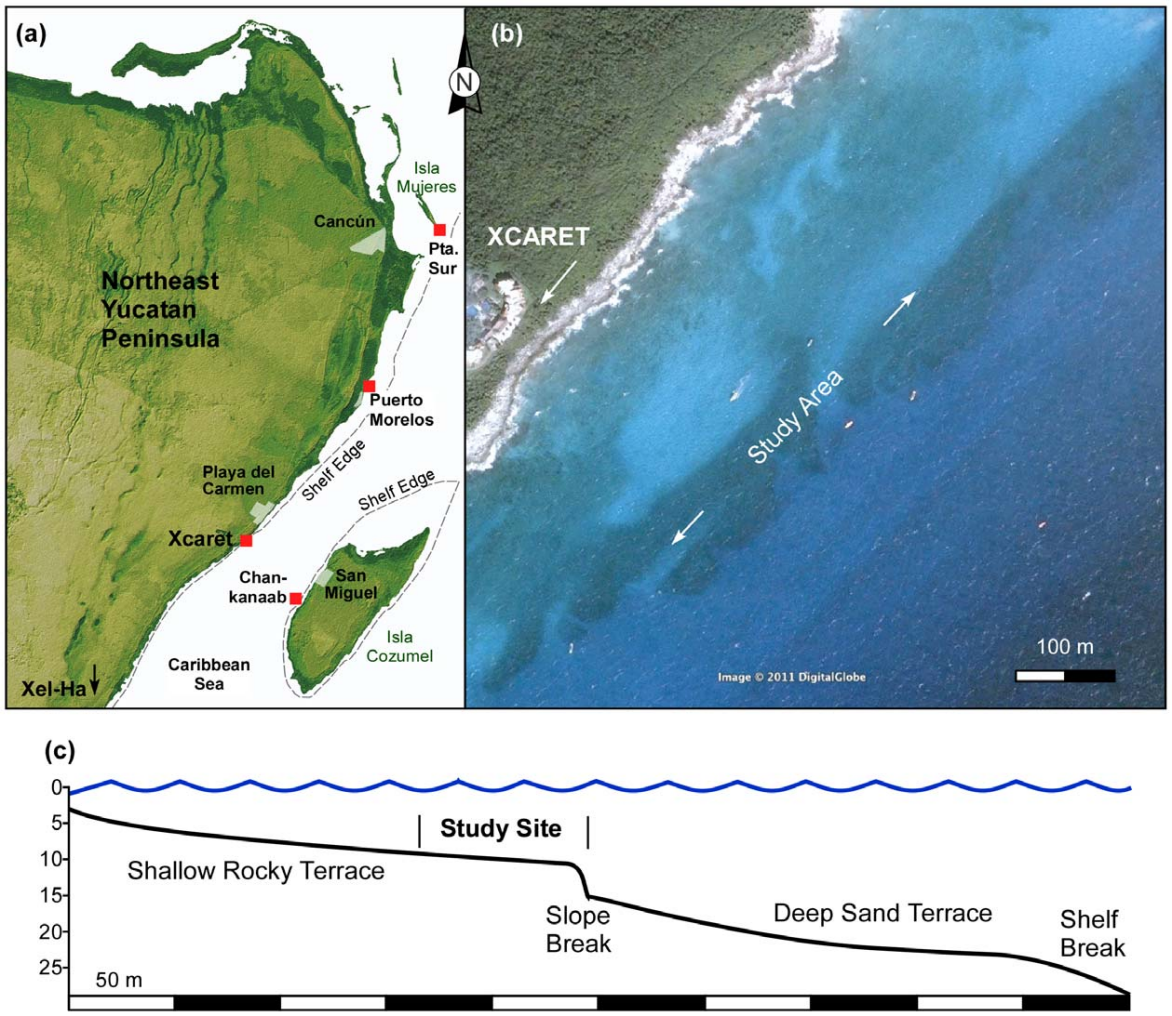
Location of the study site (a) and depth profile and zonation (b).

The coral-ground at Xcaret is similar in species richness, density and cover to coral-grounds reported from both reefal and non-reefal areas in the region [6]. For example, coral-grounds have been reported in areas where breakwater reefs are absent along the narrow leeward shelves off Cozumel (Chankanaab) and Isla Mujeres (Punta Sur), and south from Xcaret to Xel-ha on the mainland [6], [27], [28] (Figure 2a). In all these localities, the coral assemblages develop along the edges of bedrock terraces. Their coral species richness is also similar to Xcaret, but the assemblages show some variation. For example, 23 coral species have been reported for Chankanaab [29] and Xcaret (Table S1), but only 16 of them are shared. However 90% of the colonies at Chankanaab had diameters less than 20 cm, coral cover was low (3.2±0.3%) and dominant species (*P. astreoides*, *S. radians* and *M. cavernosa*) were also sediment tolerant or opportunistic brooders [29]. A similar coral-ground has been reported off Puerto Morelos, further to the north (Figure 2a). Again this community is developed on the edge of the rock terrace, but is adjacent to 1–2 km wide sand terrace. It has a low scleractinian cover (3.1±12%) and species richness (20 species) like Xcaret (5.9±2.2% and 23 species respectively). It is also dominated by small colonies of sediment-tolerant species, particularly *M. cavernosa* and *S. siderea* [30], [31].

The coral-grounds at Xcaret and other sites along the coast clearly have similar characteristics: they are located on the edge of the bedrock terrace adjacent to a slope break and they are dominated by young, sediment-tolerant, coral communities in which large old colonies are rare. Two processes therefore seem to be prevalent in controlling these communities: periodic physical removal of large colonies and a restriction of community composition and colony age due to an elevated-sediment flux.

**Figure 3:**
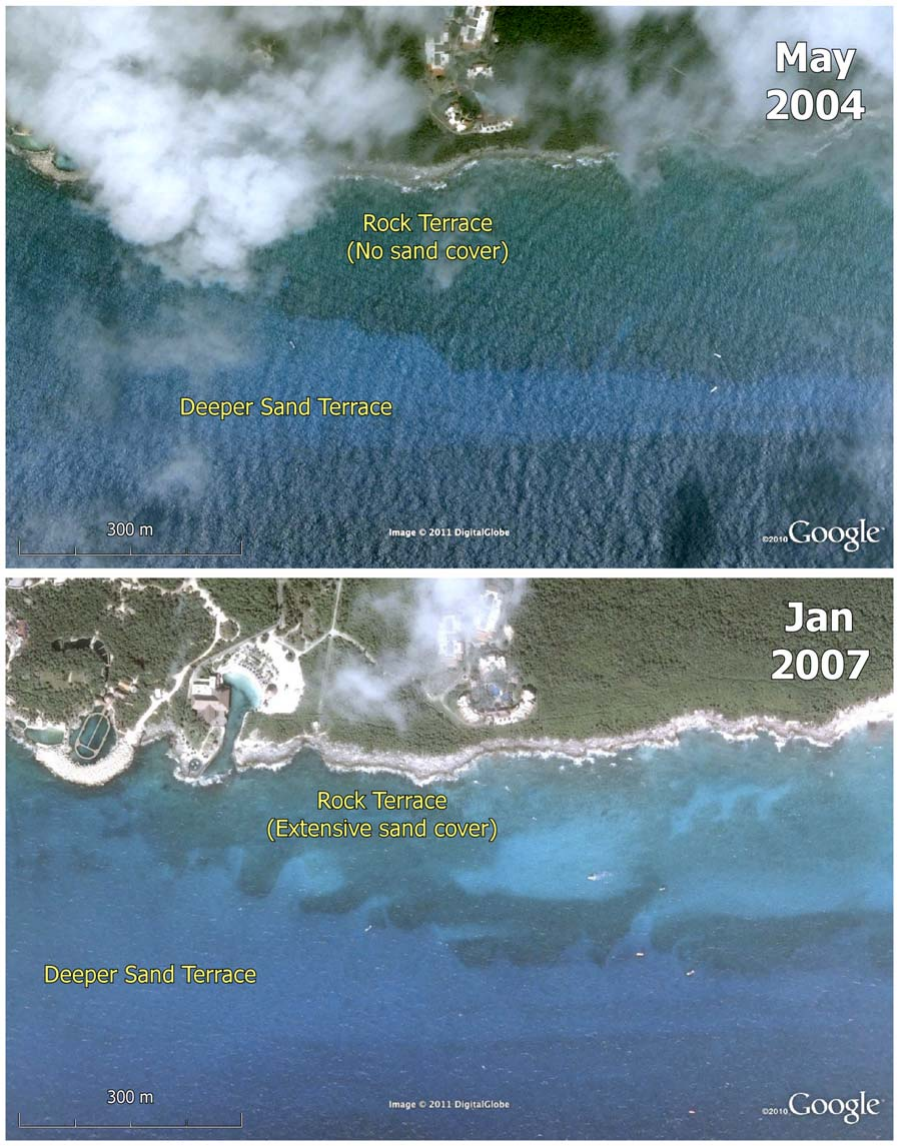
Satellite images of Xcaret showing sediment flux. Sequential satellite images showing the study site at Xcaret and a sediment-free rock terrace in May 2004, but with extensive sand cover during January 2007.

Rareness of large corals has commonly been attributed to removal during tropical cyclones [32], which are frequent in the area (see http://csc.noaa.gov/hurricanes). Wave sizes generated during these storms commonly exceed 10 m [33] and plunge and break in the depth range inhabited by the coral grounds (cf. [34]). Wave breaking during storms might therefore be responsible for physically removing the colonies above a certain size [32]. Periodic storm disturbance could also explain the ecological character of the coral community. For example, brooding species with high recruitment rates, such as *A. agaricites* and *P. astreoides* [35], [36], are known to be the first scleractinian species to recruit on disturbed reefs [23], [37].High sediment flux during storms might also restrict the development of the coral assemblage to the edge of the rock terrace adjacent to the slope break. This ‘edge effect’ results from the fact that slope breaks are less likely to be buried by sediment deposits or impacted by bedload transport during storms compared to the lower-gradient parts of the terrace [38], [39]. Indeed, satellite images from Xcaret, show inner parts of the rock terrace covered by blankets of mobile sediment that move down the coast from Playa del Carmen during North winds (Figure 3).

Although physical coral removal and elevated sediment flux during high-energy wave events is consistent with the community characteristics and location, storms and hurricanes are common along the entire Mesoamerican Reef and therefore cannot be the primary cause in preventing reef-framework development at these sites. An additional factor at Xcaret, however is the narrow shelf width, which has been shown elsewhere to prohibit reef development [34]. Around Grand Cayman, for example, breakwater reefs do not develop where the rock terrace is less than the distance that hurricane waves can carry large coral clasts (∼250 m), because clasts are thrown ashore rather than accumulating to form the foundation for reef growth [34]. The rock terrace at Xcaret is generally 250 m or less and so, if the width hypothesis is valid, it may be unsuitable for reef development. Reef absence in turn means that sediment cannot be impounded by a lagoon during storms and can freely move freely across the flat terrace surface at regular intervals, smothering incipient reef-building communities. Only at the terrace edge are corals protected from sediment smothering allowing coral grounds to develop.

In summary, the coral-ground community at Xcaret lacks large, old corals and is dominated by small, sediment-tolerant and brooding species which suffer high rates of mortality due to interactions with turf-algal sediment mats. These attributes are consistent with physical removal and high sediment flux during storms. The community only survives these conditions due to its slope-break location, which ensures lack of burial and continued local recruitment. We hypothesize that the narrow width of the rock terrace likely prevents the permanent accumulation of sediment, thereby ensuring that communities cannot escape bottom effects and develop into three-dimensional reef structures. The fact that diverse coral-ground communities exist both within and between Caribbean reef tracts therefore implies that three-dimensional reef development is not just a simple balance between accretion and erosion, but instead has specific substrate requirements determined by the geomorphology and sediment dynamics of the shelf.

The hypothesis that reef development has specific substrate requirements is testable because it predicts that coral grounds should be largely restricted to narrow rocky shelves where reef tracts are absent and sediment flux is high. It also predicts that where reefs are present, and trap sediment in their lagoons, the reduction in sediment flux should allow corals growing along shelf slope-breaks to develop into framework and produce submerged reef structures. As a consequence, future work on coral grounds should consider substrate geomorphology as a fundamental control.

## Materials and Methods

### Study area

The coral-ground studied is located on the insular shelf fronting Xcaret (20.58°, 87.12°), a tourist park 7 km south of Playa del Carmen in the NE Yucatan Peninsula, Mexico (Figure 2a). In this area the insular shelf is narrow (∼500 m), and starts from the rocky shore (Figure 2b) where a small coastal cliff descends to 2 m below sea level. At the cliff base, the seabed flattens into a narrow (∼250 m) rock terrace that gradually deepens with a slope of 25° to a depth of 10 m. The terrace is a flat, largely barren, bedrock substrate that has been sculptured by wave scour and is similar to terraces reported elsewhere in the Caribbean [40]. The coral ground is located on the edge of this terrace, which is marked by an abrupt slope break or scarp that descends from 10 m to *ca.* 12–13 m (Figure 2c). In some areas, the coral-ground community extends down to the slope break, especially where it is sub-vertical. In others, the break is steeper and forms a scarp that is indented by channels or overhangs to form small caves. At its base, the scarp flattens into an outer sand-covered terrace that slopes gently to 25 m (Figure 2c). This terraced shelf configuration is common in the Caribbean and in other areas with significant reef development and is related to variation in the rate of Holocene sea level rise [40], [41].

In other locations along the coast, the bedrock terrace has been confirmed to be composed of late Pleistocene limestone that has been leached and subaerially altered changing some of the original aragonitic mineral phase to calcite (Blanchon unpublished core data). At Xcaret, although no core data are available, the bedrock terrace was temporarily exposed in a trench cut for an aquarium outfall, and is composed of the same leached and subaerially altered limestone seen on the adjacent rocky coast [42]. This evidence of subaerial exposure proves the bedrock is a Pleistocene limestone, not a Holocene reef deposit. In addition, the bedrock terrace also shows widespread signs of wave scour and marine ravinement with erosional sculpturing and coastal cliffing into the onshore reef deposits, which have been dated as last Interglacial in age [43]. Thus, despite significant framework development during the last Interglacial in the area, and the common presence of many reef-building species elsewhere along the coast, there is no active framework accretion on the shelf at Xcaret today, nor has there been during the Holocene.

### Survey method

In collaboration with Park staff, the site survey was conducted from May to July 2005 using video-transects and photography. Ten 30×1 m belt transects, centred on a measuring tape, were haphazardly positioned perpendicular to the edge of the terrace at approximately 10–13 m depth over a linear distance of 400 m (Figure 2b). The distance between consecutive transects ranged from 5 to 30 m. All Scleractinian colonies within the belt-transect were identified to species level *in situ* and inspected for coral diseases and competitive interactions (whenever an organism touched or encroached the border of the coral colony) with macroalgae, calcareous coralline alga, turf-algal sediment mats, sponges and ascidians. Close-up photographs, with a scale, were taken of every coral colony and later analyzed to measure its size and partial mortality using the program SigmaScan Pro Version 4.0 (SPSS, Chicago, IL). Each colony was defined as any autonomous coral skeleton with living tissue, including those that were divided by partial mortality into separate patches of living tissue, but morphologically still one entity [44]. Ten transects were found to be an adequate sample size based on performance of species-area curves (i.e. cumulative species versus number of sites) levelling-off after eight transects.

Coral diseases were assigned to one of four categories: white-plague (WP), yellow-band (YB), dark-spot (DS) and black-band (BB). The percent number of colonies with diseases and competitive interactions was calculated for all Scleractinians. Data are presented as means ± standard deviation.

Bottom cover by benthic groups (coral, fleshy algae, calcareous algae, turf algae, sponges, and abiotic substratum) was obtained using video-transects [45]. The video-transect was centred on the measuring tape and filmed from a distance of 40 cm above the reef substratum, using a digital video camera (model Sony DSC-10) and housing. The camera to surface distance was controlled by a projecting aluminium rod that ended in a horizontal scale. Transect width was 0.3 m and image resolution was in the order of 0.5 cm. The video was divided into ∼80 non-overlapping photographic frames per transect that were analyzed using the software program Coral Point Count with Excel Extension (CPCe; [46]). The percent cover of benthic groups was calculated by counting 30 random points per frame. Multiple frames were combined into a single transect dataset that were analyzed for population estimates [46].

A single member of the dive team identified *in situ* the presence of non-scleractinian benthic fauna in the area to the lowest possible taxonomic level following Bayer [47] for Gorgonians and Humann [48] for the orders Actinaria and Zoanthiniaria and for the Class Ascidiacea. Taxonomic identification of sponge species was done by a member of the Xcaret aquarium through spicule and tissue preparations based on Hooper [49].

Coral colony number and partial mortality were analyzed for differences between size classes using a one-way analysis of variance with transects as replicates and size class as factor, followed by post-hoc analyses (Tukey HSD). Data were checked for homogeneity of variances with Leven's test and for normality using normal probability plots. Coral disease prevalence and competitive interactions data were analyzed using non-parametric Kruskal-Wallis tests.

## Supporting Information

Table S1 **Summary of the number of coral species, mean bottom coverage (%) and dominant coral species in terms of the total number of coral colonies sampled in three coral grounds and three coral reefs in the Mexican Caribbean.**
(DOC)
