# Supplementary material for: Controls on Coral-Ground Development along the Northern Mesoamerican Reef Tract
Source: PLoS One. 2011 Dec 14;6(12):e28461. doi: 10.1371/journal.pone.0028461 (PMC3237443; doi:10.1371/journal.pone.0028461)
Supplement: Table S1 — Summary of the number of coral species, mean bottom coverage (%) and dominant coral species in terms of the total number of coral colonies sampled in three coral grounds and three coral reefs in the Mexican Caribbean. (DOC) [file pone.0028461.s001.doc]

**Table S1.** Summary of the number of coral species, mean bottom coverage (%) and dominant coral species in terms of the total number of coral colonies sampled in three coral grounds (CG) and three coral reefs in the Mexican Caribbean.

| **Country** | **CG** |  |  |  | **Reefs** |  |  |
| --- | --- | --- | --- | --- | --- | --- | --- |
| **Site** | **Xt** | **Cha^[1]^** | **PM ^[2]^** |  | **Ak ^[3]^** | **PA^[4]^** | **Xc^[5]^** |
| Year | 2005 | 1995 | 2005 |  | 2000 | 1986 | 1999 |
| Depth (m) | 10 | 6-8 | 10 |  | 13 | 8-10 | 10 |
| Sites # | 1 | 1 | 1 |  | 8 | 1 | 3 |
| Transects # | 10 | 6 | 10 |  | 74 | 5 | ND |
| Coral species # | 23 | 23 | 20 |  | 25 | 30 | 30 |
| Mean cover % (SD) | 5.9 (2.2) | 3.2 (0.3) | 3.1 (1.2) |  | Max: 24.5 (17.0) | 24.0 (ND) | 24.3 (ND) |
| Dominant species  (contribution to N) | *Aaga,*  *Past,*  *Ssid*  (62%) | *Past,*  *Srad,*  *Mcav*  (53%) | *Mcav,*  *Ssid*  (61%) |  | *Mfav,*  *Mann*  (ND) | *Mann s.c..*  *Aten,*  *Ppor*  (ND) | *Mann s.c.*  (50%) |

Xt: Xcaret, Cha: Chankanaab, PM: Puerto Morelos, Ak: Akumal, PA: Punta Allen, Xc: Xcalak, Aaga: *Agaricia agaricites*, Past: *Porites astreoides*, Ssid: *Siderastrea* *siderea*, Srad: *S. radians*, Mcav: *Montastraea cavernosa*, Mfav: *M. faveolata*, Mann: *M*. *annularis*, s.c.: species complex, Aten: *A. tenuifolia*, Ppor: *P. porites*. ND: no data.

1. Jordán-Dahlgren E, Rodríguez-Martínez RE (1998) Caracterización de la comunidad coralina del Parque Chankanaab. In: Jordán-Dahlgren E , editor. Ecología del Ambiente Marino del Parque Chankanaab. Fundación de Parques y Museos de Cozumel A.C. – ICMyL, UNAM, p 62.
2. Rodríguez-Martínez RE, Ruíz-Rentería F, van Tussenbroek B, Barba-Santos G, Escalante-Mancera E et al. (2010) Environmental state and tendencies of the Puerto Morelos CARICOMP site, Mexico. Rev Biol Trop 58: 23-43.
3. Roy RE (2004) Akumal’s reefs: Stony coral communities along the developing Mexican Caribbean coastline. Rev Biol Trop 52: 869-881.
4. Jordán-Dahlgren E, Martín-Chávez E, Sánchez-Segura M, González de la Parra A (**1994)** The Sian Ka' an Biosphere Reserve Coral Reef System, Yucatán Peninsula, México. Atoll Res Bull 423:1-31.
5. Steneck RS, Lang JC (2003) Rapid assessment of México’s Yucatán Reef in 1997 and 1999: Pre- and Post- 1998 mass bleaching and hurricane Mitch (Stony corals, algae and fishes). Pp.294-317 In: J.C. Lang (ed.), Status of Coral Reefs in the western Atlantic: Results of initial Surveys, Atlantic and Gulf Rapid Reef Assessment (AGRRA) Program. Atoll Res Bull 496.
